# Deletion of the primase-polymerases encoding gene, located in a mobile element in *Thermus thermophilus* HB27, leads to loss of function mutation of *addAB* genes

**DOI:** 10.3389/fmicb.2022.1005862

**Published:** 2022-12-01

**Authors:** Carlos Verdú, Patricia Pérez-Arnaiz, Ana Peropadre, José Berenguer, Mario Mencía

**Affiliations:** ^1^Department of Molecular Biology, Faculty of Sciences, Centre for Molecular Biology, Universidad Autónoma de Madrid-Consejo Superior de Invesigaciones Científicas (UAM-CSIC), Madrid, Spain; ^2^Department of Biology, Faculty of Sciences, Autonomous University of Madrid, Madrid, Spain

**Keywords:** PrimPol, *Thermus thermophilus*, AddAB, comet assay, bacterial transformation, DNA repair

## Abstract

DNA primase-polymerases (Ppol) have been shown to play active roles in DNA repair and damage tolerance, both in prokaryotes and eukaryotes. The ancestral thermophilic bacterium *Thermus thermophilus* strain HB27 encodes a Ppol protein among the genes present in mobile element ICETh2, absent in other *T. thermophilus* strains. Using different strategies we ablated the function of Ppol in HB27 cells, either by knocking out the gene through insertional mutagenesis, markerless deletion or through abolition of its catalytic activity. Whole genome sequencing of this diverse collection of Ppol mutants showed spontaneous loss of function mutation in the helicase-nuclease AddAB in every *ppol* mutant isolated. Given that AddAB is a major player in recombinational repair in many prokaryotes, with similar activity to the proteobacterial RecBCD complex, we have performed a detailed characterization of the *ppol* mutants in combination with *addAB* mutants. The results show that knockout *addAB* mutants are more sensitive to DNA damage agents than the wild type, and present a dramatic three orders of magnitude increase in natural transformation efficiencies with both plasmid and lineal DNA, whereas *ppol* mutants show defects in plasmid stability. Interestingly, DNA-integrity comet assays showed that the genome of all the *ppol* and/or *addAB* mutants was severely affected by widespread fragmentation, however, this did not translate in neat loss of viability of the strains. All these data support that Ppol appears to keep in balance the activity of AddAB as a part of the DNA housekeeping maintenance in *T. thermophilus* HB27, thus, playing a key role in its genome stability.

## Introduction

DNA replication is universally accompanied by DNA repair or damage-tolerance mechanisms that are essential to preserve the fidelity of the copies in the progeny. DNA replication is carried out by dedicated replicative DNA polymerases that depend for DNA synthesis initiation on primases (Frick and Richardson, 2001). In Proteobacteria the replicative DNA polymerase can initiate DNA replication from an RNA primer synthesized by a dedicated enzyme (DnaG; Bergsch et al., 2019), but replicative DNA polymerases of other bacterial phyla may extend a RNA–DNA hybrid or directly a DNA primer. In Firmicutes, DnaG and a specialized DNA polymerase (DnaE3) synthesize a hybrid RNA-DNA primer to be extended by the replicative DNA polymerase PolC (as in the case of the eukaryotic alpha polymerase hybrid primer, that is used by the leading- and lagging strand DNA polymerases) or a new family of enzymes [Primase-polymerases (Ppol)] has the ability to start DNA chains with dNTPs (Lipps, 2003; Garcia-Gomez et al., 2013; Guilliam et al., 2015). These Ppol enzymes belong to a superfamily of archaeo-eukaryotic primases (AEPs) (Iyer, 2005; Kazlauskas et al., 2018) that have been also found in bacteria and viruses. Interestingly, the range of functions that have been proposed for AEPs goes further than primase activity and includes in prokaryotes, base excision repair (Płociński et al., 2017; Brissett et al., 2020) and defense against exogenous DNA (Zabrady et al., 2021), and, in eukaryotes, damage tolerance mediated by repriming (Mourón et al., 2013; Bainbridge et al., 2021) and mitochondrial DNA maintenance (Garcia-Gomez et al., 2013; Bailey and Doherty, 2017; Torregrosa-Munumer et al., 2017). Ppol-type genes are often found within defense genetic islands or associated to mobile genetic elements (Kazlauskas et al., 2018) suggesting that their function is also related to that of those clusters.

The thermophilic strain *Thermus thermophilus (T.th.)* HB27 (HB27 hereon) encodes a thermostable Ppol well characterized biochemically (Picher et al., 2016), and commercially used in kits for whole genome isothermal MDA amplification in combination with the Phi29 DNA polymerase (TruePrime amplification, 4BasesBio). The gene encoding this Ppol is located within the mobile genetic element ICETh2, inserted between positions 641,829 and 653,145 of the chromosome of HB27 at the 3′ end of a Val-tRNA gene (Baquedano et al., 2020). This genetic element encodes a site specific insertion-excision system that allows the element to excise as a 11.3 kbp circular element at low frequencies (10^–4^–10^–5^) under normal growth conditions. The integration system is also functional, being able to catalyze its site specific *in trans* insertion into a plasmid, being the tyrosine recombinase (Int2) encoded by ICETh2 required for both excision and integration activities. It is interesting to highlight that Int2 is also required for excision and integration into an Ile-tRNA gene (positions 1,778,501 to 1,793,358) of ICETh1, another mobile element which encodes a DNA translocation system involved in conjugative DNA scavenging (Blesa et al., 2020). It was also shown that the excision capability of these elements increase their transferability by conjugation respect to chromosomal genes, leading us to hypothesize that Ppol from ICETh2 could provide replicative functions for both elements (Baquedano et al., 2020), as suggested for putative Ppol proteins of Archaeal and bacterial mobile genetic elements (Kazlauskas et al., 2018). However, mutants lacking Ppol were not apparently affected in the copy number of the circular forms of ICETh2 or ICETh1 (Baquedano et al., 2020), suggesting a different physiological role for this Ppol protein.

Among the phenotypic traits assayed for Ppol defective mutants the most prominent effect was associated to a dramatic increase of 2–3 orders of magnitude in the efficiency of its natural competence (García-Quintans et al., 2020), a property previously found only for mutants lacking the Argonaute (Ago) protein (Swarts et al., 2014), thus supporting a putative relationship between the functions of both proteins, likely with Ppol being involved in providing Ago with the single-stranded DNA (ssDNA) guides needed for interference against incoming DNA. However some differences were found between the phenotypes of single mutants lacking Ppol or Ago, especially in relation with transformation with linear DNA designed for integration by double recombination, suggesting additional roles for Ppol or the putative involvement of additional genes in the phenotypes analyzed (García-Quintans et al., 2020).

In this work we have analyzed in greater detail different *ppol* mutants characterizing them by whole genome sequencing. We have found that absence of Ppol or inactivation of its catalytic capacity produces systematically secondary loss-of-function mutations in the AddAB helicase-nuclease, suggesting that Ppol is crucial for cell viability, and its elimination has to be compensated by mutation of a central recombination player as it is AddAB. The AddAB complex, in many bacteria, plays a role similar to that of *Escherichia coli* RecBCD (Amundsen and Smith, 2003; Gurung and Blumenthal, 2020), providing the ssDNA overhanging extensions needed for loading of the recombinase RecA (Yeeles and Dillingham, 2010) to achieve the recombinational repair of dsDNA breaks.

The data we present here suggest that Ppol is deeply imbricated in HB27 genome stability routes. Specifically, Ppol seems necessary to counteract AddAB activity in such a way that in the absence of Ppol, AddAB is toxic for the bacterium, and, mutants defective in the corresponding genes are spontaneously selected. Since the strains are viable and do not have severe DNA repair defects, alternative recombinational repair pathway/s must be acting. Still the genomes of the mutants seem to suffer great fragmentation when analyzed by comet assay.

## Results

### Whole-genome sequencing of strains reveals additional mutations

**FIGURE 1:**
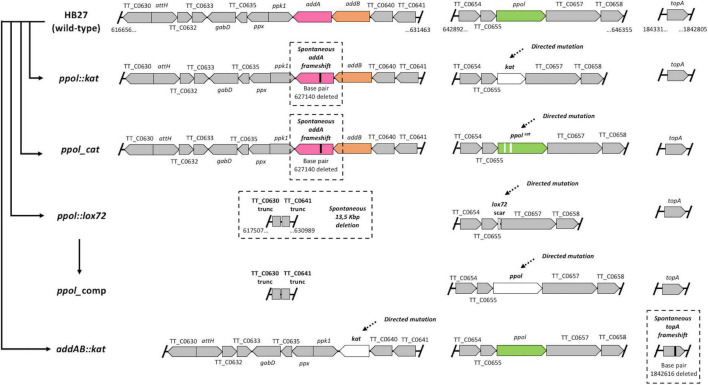
Schematic overview of all strains tested in this work. Outline of the genomic context of *ppol* and *addAB* in *Thermus thermophilus* HB27. Individual genes are indicated as well as the genomic coordinates of the ends. Integrity of the topA gene is also shown. Mutations generated are designated in white with dashed arrows and major resultant compensatory mutations are in black with dashed squares. The order of construction among strains is indicated with solid arrows. Strains presented are HB27 (Wild type), *ppol:kat* [knockout of *ppol* by insertion of a kanamycin-resistant marker (*kat*) through homologous recombination], *ppol_cat* [removal of primase-polymerases (Ppol) catalytical activity by the generation of D70A and D72A residues substitution with CaldoCas9 system], *ppol:lox72* (markerless deletion of *ppol* through Cre–lox deletion system), *ppol_comp* (restoration of ppol in ppol:lox72 strain with CaldoCas9 system), *addAB:kat* (*addAB* knockout by *kat* insertion through homologous recombination). The gene denominations as well as genomic coordinates for HB27 chromosome are shown.

In previous work we characterized a markerless *ppol* deletion mutant of the strain HB27 (*ppol:lox72* hereon) (García-Quintans et al., 2020). One of the conclusions of that study was that the *ppol* mutant had a 2–3 orders of magnitude increased natural transformation ability, using circular plasmids, linear constructs or chromosomal DNA. Otherwise, the mutant had essentially wild-type phenotype even in the presence of DNA damage agents. We performed whole genome sequencing of the mutant, using Illumina technology, to check the presence of possible additional changes in its genome, composed of a chromosome of 1.9 Mb and a megaplasmid of 232 kbp. Upon sequencing, we found a deletion of around 13.5 kbp (13 473) at a distance of 12 Kb from the *ppol* locus (Figure 1 and Table 1), in the chromosome. This deletion led to the elimination of 11 genes and truncation of 2 more (see Supplementary Table 1). Among the eliminated genes there were the ones coding for the proteins of the AddAB helicase-nuclease complex, a two component response regulator and the polyphosphate synthase and hydrolase. Additionally, at other regions of the genome, a frameshift in the *mut*S2 gene at position 496 (out of 745 aas), and a change leading to Leu442-Pro in the *recG* gene (770 aas) were found (see Supplementary discussion). It is interesting to note here that MutS2 has been shown to act as an inhibitor of homologous recombination (Pinto et al., 2005; Fukui et al., 2008). Notwithstanding, the fact that AddAB complex is a major player in recombinational genome repair in bacteria and in defense against eDNA (Simmon and Lederberg, 1972; Wigley, 2013), prompted us to design a series of mutants to elucidate the effect of the loss of Ppol alone or in combination with AddAB. The mutants constructed were the following (Figure 1):

**TABLE 1:** Comparative table of genomic changes determined by sequencing of mutant strains.

Locus/Strain	*ppol:lox72*	*ppol_cat*	*ppol_comp*	*ppol:kat*	*addAB:kat*
ppol	m.d.	D70A D72A	WT	*:kat*	WT
addAB	s.d.	AddA 311 f.s.	s.d.	AddA 311 f.s.	*:kat*
14 Kb region	s.d.	WT	s.d.	WT	WT
pTT27	WT	WT	WT	WT	WT
topA	WT	WT	WT	WT	Δ61 aas C-end
Other	mutS2 496 f.s.		mutS2 496 f.s.		

Affected loci are shown in columns: ppol, TT_C0656; addAB, TT_C0638 TT_C0639; 13 Kb region, from TT_C0635 to TT_C0641; pTT27, from TT_P0001 to TTP_P0079 plus TT_P0191 to TT_P0230; topA, TT_C1931; mutS2, TT_C1282. m.d. markerless deletion, s.d. spontaneous deletion, f.s. frameshift, exc. excluding. ppol_cat, amino acid changes indicated, aas amino acids. AddA is 857 aas and MutS2, 744. Shadowed in darker gray, large deletions; in light gray, point mutations.

– A derivative of the *ppol*:lox mutant in which the *ppol* gene was reconstituted on its chromosome original locus (*ppol_comp*), keeping the rest of the strain genotype. This mutant was designed to study the effect of the *ppol* gene comparing the phenotype of *ppol:lox* (absence of Ppol) with that of *ppol*_comp (presence of Ppol).
– A mutant in the *ppol* gene, on HB27 wild-type background, in which two catalytic residues from the DNA polymerization active site were inactivated by changing Asp to Ala (D70A and D72A) (*ppol_*cat). This was generated using the CaldoCas9-CRISPR method (see section “Materials and methods”) in order to ablate Ppol function with the minimal perturbation of the genome.
– A deletion construct in which the *ppol* gene was replaced by a cassette conferring thermostable resistance to Kn (*ppol:kat*).
– A construct in which the *addAB* genes were replaced by a cassette conferring thermostable resistance to Kn (*addAB:kat*).

A summary of the relevant genotypes obtained by whole genome sequencing of all the mutants are shown in Figure 1, Table 1. We observed that all the intended mutations were produced correctly. However, in all cases in which we tried to ablate Ppol function, by either complete deletion (*ppol*:kat, of which we sequenced three independent clones, Supplementary Table 2) or catalytic inactivation (*ppol*_cat), the genomes of the resultant strains also contained spontaneous loss of function mutations in the *add*A gene due to a frameshift mutation at Ala311 (with a lenght of 857 amino acids for AddA), or, in the case of the original *ppol:lox72* mutant, by deletion of a 13 kbp genomic region including the *addAB* genes (Figure 1 and Table 1), among others.

Other mutations not easily connectable with the functions of Ppol or AddAB were also detected in the sequenced genomes (Supplementary Table 3, see Supplementary discussion). Interestingly, the *addAB:kat* mutants have a frameshift mutation at the C-terminus of the topoisomerase I gene (*topA*), eliminating the last 61 amino acids of the protein (out of 824 aas) corresponding to the last two domains, helix-turn-helix and the zinc ribbon D9. These domains have been shown to participate in interactions with ssDNA probably to direct the Topo I to the transcription bubble to relax negative supercoiling (Tan et al., 2015).

Due to the fact that in the genome of any of the mutants there are around 20 additional mutations, we have no strictly-speaking wild-type isogenic strain for each clone. Furthermore, probably it will not be possible to generate such isogenic strains, because the genetic manipulation required, will lead to new spontaneous mutations. However, given that no spontaneous mutation is common to all clones (except the commented one, addAB), except for derived strains, and that we have used independent clones to test each phenotype, we have proceeded by comparing each strain properties to those of the closest wild type or derivative, and we will delimit the results accordingly.

Therefore, once the genotypes were determined, we performed a detailed characterization of the five mutant strains of Table 1 in terms of viability, DNA damage sensitivity, transformability with plasmid and linear DNA, plasmid loss phenotype, and genomic DNA integrity.

### *addAB* deletion increases the transformability of *Thermus thermophilus*

**FIGURE 2:**
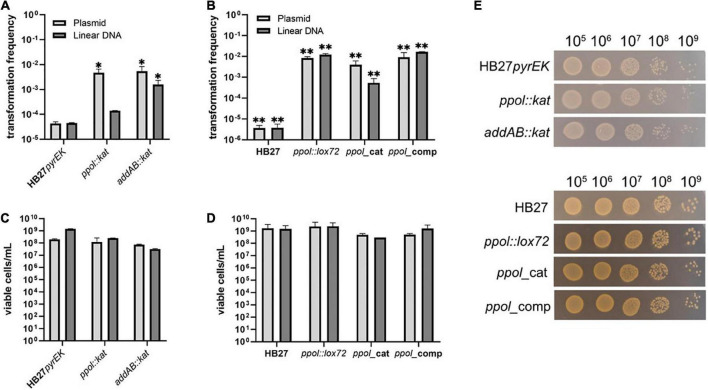
Transformation efficiency of *Thermus thermophilus* mutants. **(A)** Hundred nanogram of a replicative plasmid (pMotH1103A) or an homologous-recombination integrative linear DNA fragment conferring resistance to Hyg (pyrEH), were used to transform Tth HB27*pyrEK* control strain and *ppol:kat* and *addAB:kat* insertion mutant. **(B)** Hundred nanogram of a replicative plasmid (pMotK1103A) or an homologous-recombination integrative linear DNA fragment (pyrEK) conferring resistance to Kn, were used to transform Tth HB27 cells and its derivatives *ppol:lox72, ppol_*cat, *ppol_*comp, *addAB:lox72*, and *addAB_ppol* mutants. **(C)** Viable cells per mL of the *kat*-marked transformed strains in Kn plates. **(D)** Viable cells per mL of the markerless transformed strains in TB plates. **(E)** Serial dilutions of transformed cells of the indicated strains plated on Kn **(upper panel)** or non-selective plates **(lower panel)** and incubated for 48 h at 65°C. Transformation is represented as the number of colonies on selection plates at 65°C, relative to the number of viable colonies on non-selective plates. Transformation frequencies were calculated as an average of at least five biological replicates. Error bars correspond to the standard deviation of the means. Asterisks indicate statistically different values observed in mutant strains compared to those in the corresponding control strains (*P-value <0.005 and **P-value <0.0001).

We carried out a series of transformation experiments with the HB27 control strain and the insertion and markerless mutants. Transformation experiments were carried out with the replicative plasmids pMotK1103A and pMotH1103A, conferring thermostable resistance to kanamycin (Kn) and hygromycin (Hyg), respectively, and with linear DNA fragments to perform double recombination substitution of the *pyrE* gene with gene cassettes encoding either Kn (pyrEK) in the case of markerless mutants or Hyg resistances (pyrEH) for the Kn-resistant substitution mutants. In the case of the HB27 pyrE:kat control strain, transformations were carried out with a Hyg substitution construct for the non-essential TT_C0313 gene (ferredoxin-nitrite reductase). As shown in Figure 2A, both *ppol:kat* and *addAB:kat* deletion mutants show an increase in transformability of around 100-fold for plasmidic DNA, and 3- and 30-fold for the linear DNA respectively, as compared with the HB27 *pyrEK* control strain (we do not consider the three folds value significant enough).

Then, we tested the efficiency of transformation of the markerless *ppol:lox72*, *ppol*_cat and *ppol*_comp mutants. We also observed an increased transformability in all of them (Figure 2B). However, in these cases the multiplication factor over the wild-type value was 10^3^ for the *ppol* variants and the *addAB* mutants when transformed with linear construct or plasmidic DNA. These results suggest that the inactivation of *addAB* genes, that occurs in all the mutants tested (either complete deletion or frameshift mutation) is the main responsible for the high transformation capacity phenotype. This is based on the fact that in the *addAB*:*kat* mutant the *ppol* gene is intact and they show a clearly increased transformation ability. The effect of *ppol* deletion can be deduced comparing *ppol:lox72* with *ppol*_comp, but we do not detect significative differences. Viability of all the transformed mutant strains grown in non-selective plates was simultaneously tested, and all mutants show close to normal viability compared to the control strains (Figures 2C–E).

### *addAB* and ppol are not essential for DNA repair in *Thermus thermophilus*

**FIGURE 3:**
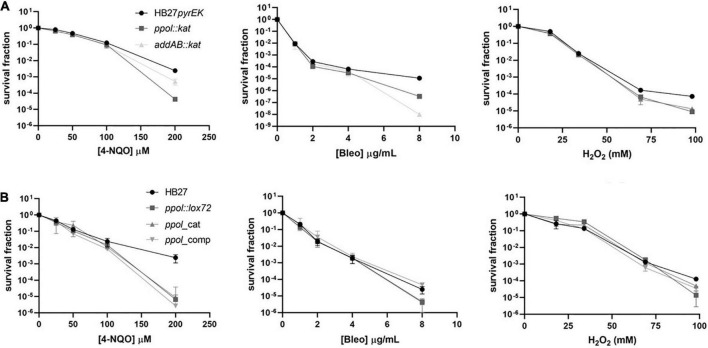
Resistance to DNA damaging agents in the *Thermus thermophilus* mutants. **(A)** The HB27*pyrEK* control strain and the *ppol:kat* and *addAB:kat* insertion mutants were subjected to different DNA damaging agents. The cell cultures were incubated at 65°C until OD_600_ of 0.3 (3 × 10^8^ cell/ml) was reached. Then, cells were further incubated for 2 h with the indicated concentrations of the different DNA damaging agents: peroxide hydrogen (H_2_O_2_), 4-Nitroquinoline-N-oxide (4-NQO) and Bleomycin (Bleo). Then, 10 μl of serial decimal dilutions were drop-inoculated on non-selective plates that were incubated at 65°C for 48 h. Fraction of surviving cells following DNA damage (4-NQO, left. Bleo, medium. H_2_O_2_, right) is calculated relative to an untreated control. **(B)** The HB27 control strain and its derivatives *ppol:lox72, ppol_*cat, *ppol_*comp, *addAB:lox72*, and *addAB_ppol* mutants were subjected to the same DNA damaging agents used in panel **(A)**. Each data point is an average of at least five biological replicates. Error bars correspond to the standard deviation of the means.

Next, we examined the sensitivity of the *T.th.* mutant strains to several chemical DNA damage agents with different effects on DNA: 4-Nitroquinoline N-oxide (4-NQO), hydrogen peroxide (H_2_O_2_), and Bleomycin (Bleo) (Figure 3). 4-NQO is a chemical compound that mimics the effect of UV radiation on DNA (Williams et al., 2010; Han et al., 2017). 4NQO-induced DNA lesions include bulky adducts on guanosines on template strand. If the Uvr(A)BC complex fails to remove the DNA distortion single-strand nicks (or gaps) are generated. The treatment with hydrogen peroxide induces oxidative damage on template bases (8-oxoguanine). It also introduces single strand nicks on the template DNA and upon replication double-strand breaks (DSB) are also generated. Bleomycin is a scission agent that inhibits DNA replication by inducing DNA strand breaks (Povirk et al., 1989).

We have already shown that the markerless *ppol* deletion mutant shows similar to wild type sensitivity to several DNA damaging agents (García-Quintans et al., 2020). The *addAB:kat* and *ppol:kat* insertion mutants were only affected at the highest doses of 4NQ-O, H_2_O_2_ and Bleo tested, with different relative sensitivities depending on the damage agent (Figure 3A) (*ppol:kat* > *addAB:kat* with 4-NQO; *ppol:kat* = *addAB:kat* with H_2_O_2_; and *ppol*_cat < *addAB:kat* with Bleo). This is in accordance to what is expected for *addAB* deletion mutants, which are more affected by Bleo-induced DNA damage, which is repaired by HR, compared by 4-NQO damage, repaired by NER.

Then, we tested the DNA damage agents on the markerless *ppol*_cat and *ppol*_comp mutants. The sensitivity of *ppol*_cat and *ppol*_comp mutants was similar compared to *ppol:lox72* and wild-type strains, being more sensitive to DNA damage only at the highest doses assayed (Figure 3B). The effect of the AddAB loss of function can be better observed in the *addAB:kat* mutant and the results show that it is comparatively more sensitive to Bleo, again as expected, because the damage would be repaired by HR. We also observed that the *ppol:kat* mutant is more sensitive than *addAB:kat* to 4-NQO at the highest dose tested, which could be ascribed to the absence of Ppol, given that both mutants presumably lack a functional AddAB complex. The mutants *ppol:lox72* and *ppol*_comp show little effect on their sensitivities to the three agents, this could be due to possible compensatory effects of the additional mutations (13 kbp deletion and MutS2 inactivation) occurring in these strains. In the case of *ppol*_cat, the point mutations present would lead to a phenotype milder than that of *ppol:kat* as there are no whole-gene deletions in this mutant.

### The genomes of *ppol* and *addAB* mutants appear as heavily fragmented by comet assays

**FIGURE 4:**
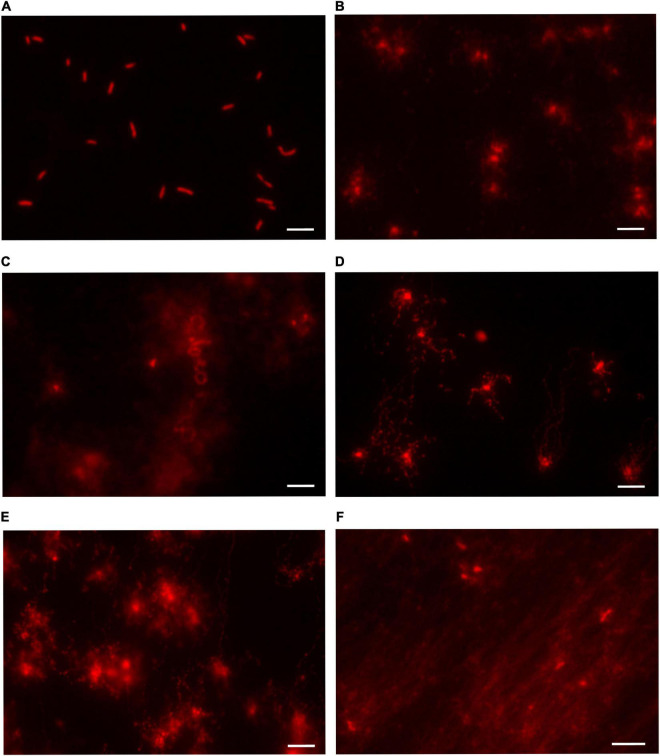
DNA integrity analysis in *Thermus thermophilus* mutants. Representative images of control and mutant strains analyzed by neutral comet assay and stained with GelRed^®^. The whole extent of the comet tail relative to the head provides qualitative information about the amount of DNA lesions **(A)** HB27. **(B)** *ppol::kat*. **(C)** *addAB::kat*. **(D)** *ppol_cat*. **(E)** *ppol::lox72*. **(F)** *ppol_comp*. In the HB27 cultures, the majority of the cells were intact showing little or no DNA migration (fluorescence limited to nucleoids). On the other hand, in the mutants DNA leaked out from the nucleoid indicating the presence of strand fragmentation. The different halos and migration patterns are indicative of the amount of DNA damage. Cells with extensive lesions had almost all DNA in the tail due to migration from the nucleoid.

From the previous results, all the mutant strains appeared to be similar to the parental HB27 in terms of viability, and their higher sensitivity to DNA damage agents was only apparent at the highest concentrations. Additionally we performed DAPI staining and microscopy visualization of samples of all the mutants plus HB27, and, again, the results (Supplementary Figure 1) showed no particular differences in nucleoid staining between HB27 and the mutants. In order to have a different overview of the integrity of the genome in these strains we performed a series of neutral comet assays. The comet assay consists in a single-cell gel electrophoresis test to visualize DNA damage. Comet’s DNA-tail provides information about the extent of DNA lesions. In this way, if the DNA is intact, it approximately keeps the shape of the nucleoid. However if the DNA is fragmented, the electrophoretic process separates and linearizes the strands creating a tail that moves away from the remains of the nucleoid. The results (Figure 4) show that for HB27 the nucleoid DNA maintains an elliptic, compact aspect, indicating that the genome has not sustained a detectable level of DNA damage. However, strikingly, in all the mutants important genome fragmentation was observed through the extension length, and diffusion pattern of the DNA. All the mutant strains seem to suffer some level of DNA damage but a qualitative order would be HB27 (no damage) < *ppol:kat* < *ppol_*cat < *ppol:lox72* = *ppol_*comp < *addAB:kat*.

### Plasmid stability in the absence of Ppol and/or AddAB

**FIGURE 5:**
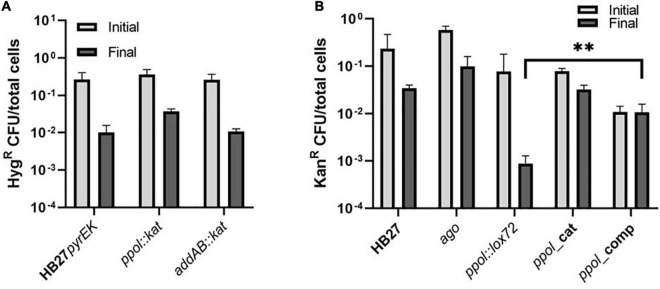
Plasmid stability in the *Thermus. thermophilus* mutants. **(A)** Comparison of plasmid loss frequencies using a replicative plasmid conferring Hyg resistance (pMotH1103A) in HB27*pyrEK* control strain and in *ppol:kat* and *addAB:kat* insertion mutant strains. Transformed cells were re-streaked twice on selective plates and grown on selective media liquid for 24 h. After that, cells were further incubated for another 48 h in the absence of antibiotic to allow plasmid loss to occur. We finally plated cells on both non-selective and selective plates and the plasmid loss frequency was measured as the CFUs on selective media divided by CFUs on non-selective plates (total cells). **(B)** Comparison of plasmid loss frequencies using a replicative plasmid conferring Kn resistance (pMotK1103A) in HB27 control strain and its derivatives *ppol:lox72, ppol_*cat, *ppol_*comp, *addAB:lox72* and *addAB_ppol* mutants. Each data point is an average of 3 independent experiments. Error bars correspond to the standard deviation of the means. Asterisks indicate significant statistical differences (**P-value <0.0002 when comparing the mean of *addAB:lox72* with the mean of *addAB_ppol*).

Then we tested if plasmid stability was somehow affected in the *ppol* and *addAB* deletion mutants (Figure 5). First, we transformed mutant and control strains with a replicative antibiotic-resistant plasmid (pMotK1103A or MotH3110A) followed by selection on the corresponding resistances. Cultures of these strains were allowed to grow for 40 h in the absence of the antibiotic to permit plasmid loss to occur (see section “Materials and methods”), and samples from each of the cultures were plated on agar plates in parallel with and without antibiotic. In 40 h around 51–53 generations of bacteria will take place and the copy number of the plasmid ranges from 4 to 10 copies per cell (de Grado et al., 1999).

We observed that insertion mutants showed a similar level of antibiotic-resistant cells, before and after incubation under non-restrictive conditions compared to the control strain (Figure 5A). The three strains showed a decrease in the number of resistant cells of around one log compared to the cells grown without the antibiotic (Figure 5A), indicating that Ppol and AddAB would not be critical for plasmid stability. Then, we tested the markerless mutants, and we also included a markerless Argonaute deletion mutant (*ago*) in this assay, since it was described that in this mutant the plasmid copy number is increased by 3 to 5-fold (Swarts et al., 2014). We also observed a higher than wild-type plasmid stability in the *ago* mutant. Interestingly, *ppol:lox72* exhibited a sharp decrease in antibiotic-resistant cells after growing under permissive conditions compared to wild type (Figure 5B). This result is in contrast to the one observed with the *ppol:kat* (Figure 5A). When *ppol* is restored in the *ppol_*comp mutant (that shares the same genotype as *ppo:lox72*, with the exception of the *ppol* gene restored) that effect is reversed and the number of resistant cells is recovered, up to the levels of cells incubated with antibiotic. This would suggest that Ppol in this genetic context is helping to maintain the plasmid. These results suggest a role of Ppol in plasmid stability only in the context of a genetic background in which other genes involved in repair and/or defense mechanisms are missing (Supplementary Table 3). The effect of *addAB* in plasmid stability is inconclusive and appears as part of the genetic context. The case of the catalytic mutant is particular, as its behavior in plasmid maintenance is similar to HB27 and *ppol:kat*, against our expectations of a higher loss of plasmid. Again the genetic context, where few other genes are affected, would allow redundant mechanisms to compensate for loss of Ppol activity in this mutant.

## Discussion

In order to elucidate the function of Ppol in *T. thermophilus* HB27, we constructed a series of mutants that either eliminated its gene, or, with minimal changes to the genome, mutated the codons for the catalytic residues of Ppol. When the genomes of these strains were sequenced, in all cases, the *addAB* genes had undergone complete deletion or frameshift mutations leading to a truncated AddA. This strongly suggests that, in HB27, Ppol function is required to balance the AddAB activity, that otherwise would be toxic for the cell. Among Ppol functions, repriming after stalled replication forks and gap filling have been reported. If any or both of those functions are missing and cannot be replaced, the predicted outcome would be the generation of ssDNA. These regions are prone to undergo breaks leading to double strand DNA ends, which constitute the optimal substrate for AddAB helicase-nuclease activity. It has also been shown that AddAB helicase activity is, in general, very processive (Yeeles and Dillingham, 2010), being checked only by recognition of Chi sequences that induce the generation of 3′ssDNA extensions that are a substrate for HR route. AddAB strong activity on an excess of dsDNA ends would be the cause of its toxicity in HB27 cells devoid of Ppol, and the reason underlying the systematic loss of function mutations on *addAB* genes whenever Ppol function has been abrogated.

The *addAB* genes have not been identified in *Deinococcus* (Lim et al., 2019), but a BLAST search finds them in a majority of sequenced *Thermus* species (152) (Supplementary Table 4). A search for the essential gene *rpoB* (beta subunit of RNA polymerase) finds it in 288 genomes but 95 are from the same strain, *T.th.* HB8, and the actual species are the same as with *addB* (but for *Thermus. islandicus*). Ppol homologs are encoded also in most strains of *Thermus* spp., however, there are at least 13 sequenced strains with recognizable *addAB* genes but without *ppol* (Supplementary Table 4). This would indicate that Ppol function is not absolutely required in *Thermus*, and, probably, it is not absolutely necessary either to balance AddAB activity. Furthermore, Ppol is encoded in a mobile element (ICETh2), a fact that suggests that it can be acquired, or lost, by *Thermus* strains with relative ease. However, apparently, in the HB27 strain, Ppol has become so functionally imbricated with the DNA maintenance systems of the cell that its absence must be compensated with the concomitant loss of AddAB, a central player in the HR route.

Derivatives of the HB27 strain missing *addAB* are viable and do not present a significantly higher sensitivity to DNA damage agents than the wild type (see Table 2 for a summary of the phenotypes of the characterized strains). However, as mentioned above, the *addAB* mutant bears a number of additional mutations that probably compensate for *addAB* deletion [prominently among them, a deletion of the C-terminal domain of *topA* (see Table 1)]. This means that *addAB* is not absolutely essential for this organism, but at the same time, we can only ascertain the viability and DNA damage sensitivity parameters in the genomic context shown in Supplementary Table 3, this is, including compensatory mutations. For most mutants, a slight increase in sensitivity is only apparent at the highest damaging agent concentration assayed. These results point out to the action of alternative and efficient DNA repair pathways in HB27 cells in the absence of AddAB/Ppol.

**TABLE 2:** Strains studied in this work and their resultant phenotypes.

Strain	Transformation efficiency		Plasmid loss	Sensitivity			Viability	DNA damage (Comet assay)
					
	Plasmid	Linear		4-NQO	Bleomycin	H_2_O_2_		
**HB27**	*	*	*	*	*	*	***	*
***ppol:kat***	**	*	*	**	**	**	***	**
***addAB:kat***	**	**	*	***	***	**	***	***
***ppol:lox72***	***	***	***	***	**	**	***	***
***ppol_comp***	***	***	*	***	*	*	***	***
***ppol_cat***	***	**	*	***	**	*	***	**

Asterisks represent qualitative information on the phenotypes.

Regarding transformation (Table 2), while, at this point, we cannot discard an effect of the absence of Ppol, most of the increase in transformation ability could be attributed to the absence of AddAB, or, alternatively, it would show an epistatic phenotype. In line with this, AddAB or its equivalent complex RecBCD have long been considered as potent defense systems against eDNA (Simmon and Lederberg, 1972; Cheng, 2020).

Plasmid loss appears as the only phenotype that could differentially be attributed to Ppol absence, with the caveat that six additional variations exist between *ppol:lox72* and *ppol*_comp, other than the presence of *ppol* in the last. Such putative Ppol function. This would be in accordance with the available information on Ppol protein. A semi-artificial replicating plasmid would produce ssDNA regions, as the replication machinery is not finely tuned to such a replicon, and these ssDNA gaps would be converted to dsDNA by Ppol action, so avoiding strand breaks and preserving the stability of the plasmid. Absence of Ppol would reduce the proportion of viable plasmid molecules, due to breaks and degradation, leading to plasmid loss.

The comet assay results are striking, since the viability of the different mutant strains is very similar to that of the wild-type in rich medium, and even in the presence of DNA damaging agents differential loss of viability in the mutants is only clear at the highest concentrations. All the mutant strains, but not the wild type, showed large halos indicating a high level of genome fragmentation. Taking into account that *T.th.* has been reported to be polyploid, with up to five copies of its genome per cell (Ohtani et al., 2010), the comet assay would suggest that, in the mutant strains, the genome is continuously being broken, repaired, probably by HR, and broken again, in a cyclic fashion. In this way the genome, as a whole, is preserved, but individual chromosomes would be fragmented at any given time. HR is probably the mechanism for genome repair, since *Thermus*, apparently, does not have a non-homologous end joining pathway, the informational content of the genome is not degraded (near normal viability), and RecA is important for genome integrity in this organism (Castan et al., 2003). The HB27 strain does have RecJ (Yamagata, 2001) and RecQ genes (Brüggemann and Chen, 2006), together with the RecFOR pathway (Chaudhary et al., 2020), and other DNA repair proteins typical of archaea, like HerA (Blesa et al., 2017). In the absence of AddAB, this set of genes could by itself support an active HR system that continuously repairs the genome and keeps its functionality, while allowing efficient transformation with plasmid and linear DNA, as can be observed in the mutant strains assayed.

There are few studies of Ppol function in bacteria. It has been shown that Prim-PolC of *Mycobacterium smegmatis* is dispensable for viability, by itself has some increase in sensitivity to the DNA damage agent cumene hydroperoxide, and, thus, it has been proposed to fill up small ssDNA gaps produced during base excision repair of DNA (Brissett et al., 2020). Ppol-like genes are frequently present in mobile elements but, in general, the functions associated to genes in those elements tend not to be essential for the corresponding organisms, otherwise the genes would be located in more stable regions of the genome, but there are probably many possible situations.

As it happens in *T.th.*, AddAB or its functional equivalent RecBCD seems to be dispensable for viability in other organisms. However, while in *E. coli* (Lloyd et al., 1987) loss of function of RecBCD leads to a marked decrease in HR, in *Bacillus subtilis* deletion of AddAB produces a slight decrease (Alonso et al., 1993), and in *Helicobacter pylori* the same loss of function yields an increase of 3-fold in HR after transformation (Marsin et al., 2010). The two orders of magnitude minimum increase in transformation frequency for both plasmid or linear DNA that we have observed in mutants lacking AddAB could be explained if we assume that in this bacterium the AddAB complex is exceptionally active and degrades most of the DNA molecules that enter the cytoplasm by natural competence.

As a working model we would postulate that in the absence of Ppol, the genome would be more prone to dsDNA breaks during replication at high temperatures. This is because, as it has been shown in other systems Ppol can prime after lessions and fill in gaps, that otherwise would left exposed ssDNA stretches leading in turn to DSBs (Mourón et al., 2013; Kobayashi et al., 2016; Quinet et al., 2020). The DSBs are the preferred substrate for the very active AddAB complex that would cause a high degree of attrition of the genome, and then, consequently, spontaneous loss of function mutations on *addAB* would systematically arise to avoid the genomic damage caused by this complex.

In conclusion we can say that loss of function mutation of HB27 Ppol leads systematically to loss of function mutation of AddAB. Mutants in *addAB* plus *ppol* or *addAB* alone are viable, although, for the moment, only in the genetic contexts reported here; they have a two to three orders of magnitude increased transformation efficiency and are still HR proficient, but, interestingly, suffer a high degree of fragmentation of their genomes as observed by comet assays. And the fact that Ppol, encoded in a mobile element, seems to balance the activity of a key HR player as AddAB, suggests that, from an evolutive point of view, this mobile DNA polymerase has got deeply integrated into the genome maintenance system of the bacterium.

## Materials and methods

### Strains and growth conditions

The strains used and isolated along this work are described on Table 1. *E. coli* was grown at 37°C under stirring in liquid Lennox Broth (LB) medium or in 2% (w/v) agar plates. *T.th.* was grown at 65°C in TB liquid medium containing 3 g of NaCl (Condalab), 4 g of yeast extract (Condalab) and 8 g of tryptone (Condalab) per liter of carbonate-rich mineral water, under shaking (180 rpm) or in 1.5% (w/v) agar plates. Kanamycin (Kn, 30 μg/ml), Ampicillin (Am, 100 μg/ml) or Hygromycin (Hyg, 50 μg/ml) were used for selection.

### Plasmids and construction and isolation of *Thermus thermophilus* mutant strains

Strains used in this work are listed in Table 3, and plasmids Table 4. The cloning and gene construction were first amplified in *E. coli DH5*α and then transferred to *T.th*. DNA manipulation and cloning were performed using standard laboratory techniques. All constructs were checked by restriction analysis and sequencing, and mutants were confirmed by PCR analysis. Genomic DNA for PCR and NGS sequencing was prepared using DNeasy UltraClean Microbial Kit (QIAGEN).

**TABLE 3:** Strains used in this work.

Strain	Genotype	Phenotype/Use	Reference/Source
*E. coli* DH5α	*supE4 ΔlacU169 (ϕ80 lacZΔM15) hsdR17, recA1, endA1, gyrA96, thi-1 relA1*	Cloning	
*Thermus thermophilus* strains			
HB27	*ATCC BAA-163/DSM7039*	wild type	Koyama et al., 1986
HB27*pyrE*	*ΔpyrE:kat*	Kn^R^, Chromosome labeled	García-Quintans et al., 2020
HB27*ago*	*Δago*	Hypercompetent	Blesa et al., 2015
HB27*ppol:lox72*	*Δppol::lox72, Δ617578-631463*, see Suplementary Table 3	Hypercompetent	García-Quintans et al., 2020
HB27*ppol:kat*	*Δppol:kat, addA*, see Supplementary Table 3	Kn^R,^ Hypercompetent	García-Quintans et al., 2020
HB27 *ppol_*cat	*ppol*^cat^*, addA*, see Supplementary Table 3	Hypercompetent	This work
HB27 *ppol*_comp	*Δ 617578-631463*, see Supplementary Table 3	Hypercompetent	This work
HB27 *addAB:lox72*	*ΔaddAB::lox72, ΔpTT27*, see Suplementary Table 3	Hypercompetent	This work
HB27 *addAB:kat*	*ΔaddAB:kat*, see Supplementary Table 3	Kn^R,^ Hypercompetent	This work
HB27 *addAB_ppol*	*ΔaddAB:lox72, Δppol:lox72*, see Supplementary Tables 3, 4	Hypercompetent	This work

**TABLE 4:** Plasmids used this work.

Plasmid	Description/Use	References
pMotK1103A	Bifunctional modular vector, *kat*	Verdu et al., 2019
pMotH1103A	Bifunctional modular vector, *hyg*	Verdu et al., 2019
pUC19	Cloning in *Eco* of construct for deletion mutants, Amp^R^	Vieira and Messing, 1982
pyrEK	Suicide plasmid in *T.th*. Amp^R^ (*Eco*), Deletion of pyrE gene with *kat*	García-Quintans et al., 2020
pyrEH	Suicide plasmid in *T.th*. Amp^R^ (*Eco*), Deletion of pyrE gene with *hyg*	García-Quintans et al., 2020
pUC19:*TTC0313*:*hyg*	Suicide plasmid in *T.th*. Amp^R^ (*Eco*), Deletion of TT_C0313 gene with *hyg*	Álvarez et al., 2014
pD2lox	Suicide in *T.th*. Deletion of *ppol* by insertion of *kat* flanked by *lox* sites	García-Quintans et al., 2020
pUC19:*addAB*:*kat_lox*	pUC19:*addAB*:kat. Suicide in *T.th*. Deletion of *addAB* by insertion of *kat* flanked by *lox* sites	This work
p174Cre	Bifunctional. Expression of Cre in *T.th.*, *hyg*	García-Quintans et al., 2020
pTTCC	Shuttle vector for CaldoCas9 expression in *T. thermophilus*	Adalsteinsson et al., 2021
pTTCC_Ppol	pTTCC with a *ppol*-targeting guide cloned for *ppol *^cat^** insertion in the genome	This work
pTTCC_Comp	pTTCC with guide for *ppol* insertion in *ppol:lox72* strain	This work
pUC19_Template_CatPpol	Homologous recombination template for *ppol *^cat^** CRISPR-insertion in wild type strain	This work
pUC19_Template_CompPpol	Homologous recombination template for *ppol* CRISPR-insertion in *ppol:lox72*	This work

*Thermus thermophilus* insertion knockout mutants were constructed by double recombination with a linearized DNA construct containing 1 kbp-long upstream and downstream flanking region around the target gene separated by the *kat* gene cassette, encoding thermostable resistance to Kn. In all cases, the cassette was inserted in the same transcription sense as the target gene to allow the expression of downstream gene. Resistant clones were re-streaked twice on selection plates to avoid the presence of wild-type copies of the targeted gene since *T.th.* is a polyploid bacteria to finally obtain Δ*gene:kat* mutant.

### CRISPR genome edition with CaldoCas9

Genome edition in *T.th.* with CaldoCas9 was adapted from Adalsteinsson et al. (2021). A total of 30 nt long ssDNA oligonucleotides containing spacers were designed with 4 nt long overhangs at 5′-end, complementary to *Bpi*I (ThermoFisher Scientific; ref: ER1011) digested pTTCC. Top-strand oligonucleotides contained a 5′-TGGA-3′ overhang, whereas complementary bottom-strand oligonucleotides contained a 5′-TGAC-3′ overhang. Top and bottom strands were phosphorylated with T4 polynucleotide kinase (ThermoFisher Scientific; ref: EK0031) and hybridized in a temperature gradient from 95 to 10°C. Resulting dsDNA fragments and *Bpi*I-digested pTTCC were purified with Wizard^®^ SV Gel and PCR Clean-Up System (Promega; Ref: A9281) and ligated with T4 DNA ligase (Promega; Ref: M1801). After ligation, reactions were transformed in chemically competent *E. coli* DH5α and plated in LB – kanamycin 30 μg/ml. Homologous recombination template (HRT) was designed and constructed separately from pTTCC. Corresponding oligonucleotides were used to amplify Up and Down recombination arms from *T.th.* genome and fuse them by fusion PCR (PfuUltra II Fusion HS DNA Polymerase; Ref: #600670). Resulted amplicons were digested and cloned in a pUC19 vector (suicide in *T.th.*) between *Eco*RI and *Pst*I. After cloning steps, 200 ng of both pTTCC_Guided and HRT were added to 500 μl of a 0,3 O.D._600 *nm*_ *T.th.* culture and incubated at 65°C during 3 h for natural competent transformation. Transformed cultures were plated on TB plates with 30 μg/ml of kanamycin (TBK) and incubated at 65°C for selection. Resulting colonies were two times refreshed in TBK plates in order to stabilize mutation in the genome. PCR—positive clones were curated from pTTCC refreshing in liquid TB medium with no antibiotic and plating for isolated colonies. Colonies which does not grow in TBK were checked again by PCR and stored at −80°C in 15% glycerol.

### NGS genome sequencing

The genomes of the indicated strains were sequenced by the company MicrobesNG^1^ with a target coverage of 30-fold. Reference genome for HB27 strain (NC_005835.1 and NC_005838.1) was downloaded from the NCBI. The reads of mutants strains were aligned against the reference genome using BWA aligner (Li and Durbin, 2009). Picard Tools^2^ was used to clean, sort, and mark duplicates of mapped Binary Alignment Map (BAM) files. The final BAM file was used for Variant Calling (Sandmann et al., 2017). This was performed using the GATK toolkit (McKenna et al., 2010) to identify SNPs on each mutant with the HaplotypeCaller tool. The results were processed using an in-house script written in Python language (compareSNP_betweenSamples.py) to compare all variants positions between samples and to detect the genetic location of each variant. All the sequences were uploaded to the European Nucleotide Archive^3^ under these study numbers and sample references: PRJEB42416 [HB27A lab stock (6,979), *ppol:lox72* (12,236)]; PRJEB46037 [*ppol*:*kat* (12,247)]; PRJEB53361 [*addAB:kat* (233,409), *ppol:kat*_R1 (233,415), *ppol:kat*_R4 (233,416), *ppol*_cat (232,477), *ppol*_comp (232,478)].

The tables compiling the variant postions present in the different strains were manually curated eliminating the variants occurring in our HB27 lab stock strain respect to the NCBI reference strain, and therefore they were subtracted from the analysis of all the HB27 stock derived strains.

### Transformation, DNA damage, and plasmid stability assays

*Thermus thermophilus* strains were transformed by natural competence (Koyama et al., 1986). The desired amount of DNA (100 ng) was added to 0.5 ml of mid-exponential cultures of *T.th*. After 3 h incubation at 65°C, the cells were spread on selection plates and then incubated for 2 days at 65°C. Transformation frequencies were calculated as the number of colony forming units (CFU) on selective plates divided by the number of CFUs in non-selective plates. The linear DNAs for transformation were generated by PCR from the plasmids pyrEK and pyrEH using M13 forward and reverse primers.

To study the effects of three different chemical DNA damaging agents: peroxide hydrogen (H_2_O_2_), 4-Nitroquinoline-N-oxide (4-NQO) and Bleomycin (Bleo) on the survival of *T.th.* cells, cultures at stationary phase grown at 65°C were diluted to OD_600_ −0.05 and grown on TB up to OD_600_ −0.3 at 65°C. Then, 0.5 ml of these mid-exponential cultures were further incubated with the indicated concentrations of the different DNA damaging agents for 2 h at 65°C. Then, 10 μl of serial dilutions were drop-inoculated on non-selective TB plates and further incubated at 65°C for 48 h to allow the growth of the surviving cells. The survival fraction is calculated as the fraction of colonies obtained in the treated condition respect to the untreated control.

To study plasmid stability, *T.th.* cells were first transformed with a plasmid expressing an antibiotic resistance cassette (*kat* or *hyg*). To ensure the cells are carrying the plasmid initially, they are first grown under restrictive conditions (in the presence of antibiotic) for 24 h. Then, they are maintained for another 48 h, with two dilutions to initial conditions, in the absence of antibiotic to allow plasmid loss to occur. After that, 10 μl of serial dilutions were drop-inoculated on TB plates with and without the selective antibiotic and further incubated at 65°C for 48 h. Plasmid loss frequencies were calculated as the number of CFU on selective plates divided by CFUs on non-selective plates.

### Comet assay for bacterial DNA integrity

The presence of DNA strand breaks was assessed using the neutral comet assay adapted from Singh et al. (1999) with minor modifications. An estimated 10^8^ cells were embedded in 0.5% low melting point agarose (LMPA) and deposited on pre-coated slides with 1% agarose. Immediately after agarose solidification (10 min on ice), a second layer of 0.5% LMPA solution containing 5 μg/ml RNase A (Roche), 1 mg/ml lysozyme (Roche) and 0.25% sodium N-lauroyl sarcosine was added to form a stratified microgel. The slides were refrigerated for 10 min at 4°C and incubated for 30 min at 37°C. Embedded cells were then lysed 1 h at room temperature in a buffer containing 2.5 M NaCl, 100 mM EDTA, 10 mM Tris pH 10, 1% sodium lauroyl sarcosine, and 1% Triton X-100. Following the lysis, the slides were immersed in a digestion solution [2.5 M NaCl, 10 mM EDTA, 10 mM Tris pH 7.4, and 0.5 mg/ml of proteinase K (Sigma)] for 2 h at 37°C. The DNA was allowed to unwind for 30 min in the electrophoresis buffer (300 mM sodium acetate and 100 mM Tris, pH 9), and the electrophoresis was carried out for 50 min at 0.5 V/cm. Following electrophoresis, the slides were sequentially immersed in 1 M ammonium acetate prepared in ethanol for 20 min and absolute ethanol for 30 min. Slides were allowed to dry until complete ethanol evaporation. The samples were stained with 100 μl of GelRed (Thermo Fisher Scientific) and examined with a Leica DMI 3000B microscope (Germany) equipped with an EL6000 compact light source and a 480–550 nm wide band excitation filter, and a 590 nm cut-off filter.

### DAPI staining and visualization

*Thermus thermophilus* strains were allowed to grow in rich media until stationary phase. Thereafter, 10^9^ cells were pelleted and washed with 500 μl of phosphate-buffered saline (PBS) pH 7.4 twice. 50 μl of washed cells were incubated 5 min with 0.5 μl of 4′,6-diamidino-2-phenylindole (DAPI) 0.5 μg/ml stock. After incubation, cells were washed twice with 150 μl of PBS and resuspended in the same volume. 5 μl of the final cell suspension were used for fluorescence microscope visualization at 60× magnification.

## Data availability statement

All the sequences were uploaded to the European Nucleotide Archive (https://www.ebi.ac.uk/ena/browser/home) and are publicly available under these study numbers and sample references: PRJEB42416 [HB27A lab stock (6979), *ppol::lox72* (12236)]; PRJEB46037 [*ppol::kat* (12247)]; PRJEB53361 [*addAB::kat* (233409), *ppol::kat_R1* (233415), *ppol::kat_R4* (233416), *ppol_cat* (232477), and *ppol_comp* (232478)].

## Author contributions

CV, AP, and PP-A performed the experimental work and wrote sections of the manuscript. MM and JB wrote the first draft of the manuscript. All authors contributed to conception and design of the study, manuscript revision, read, and approved the submitted version.

## Footnotes

1
www.microbesng.com

2
https://broadinstitute.github.io/picard/

3
https://www.ebi.ac.uk/ena/browser/home
