# Supplementary figures and images for: Deletion of the primase-polymerases encoding gene, located in a mobile element in Thermus thermophilus HB27, leads to loss of function mutation of addAB genes
Source: Front Microbiol. 2022 Dec 1;13:1005862. doi: 10.3389/fmicb.2022.1005862 (PMC9751324; doi:10.3389/fmicb.2022.1005862)

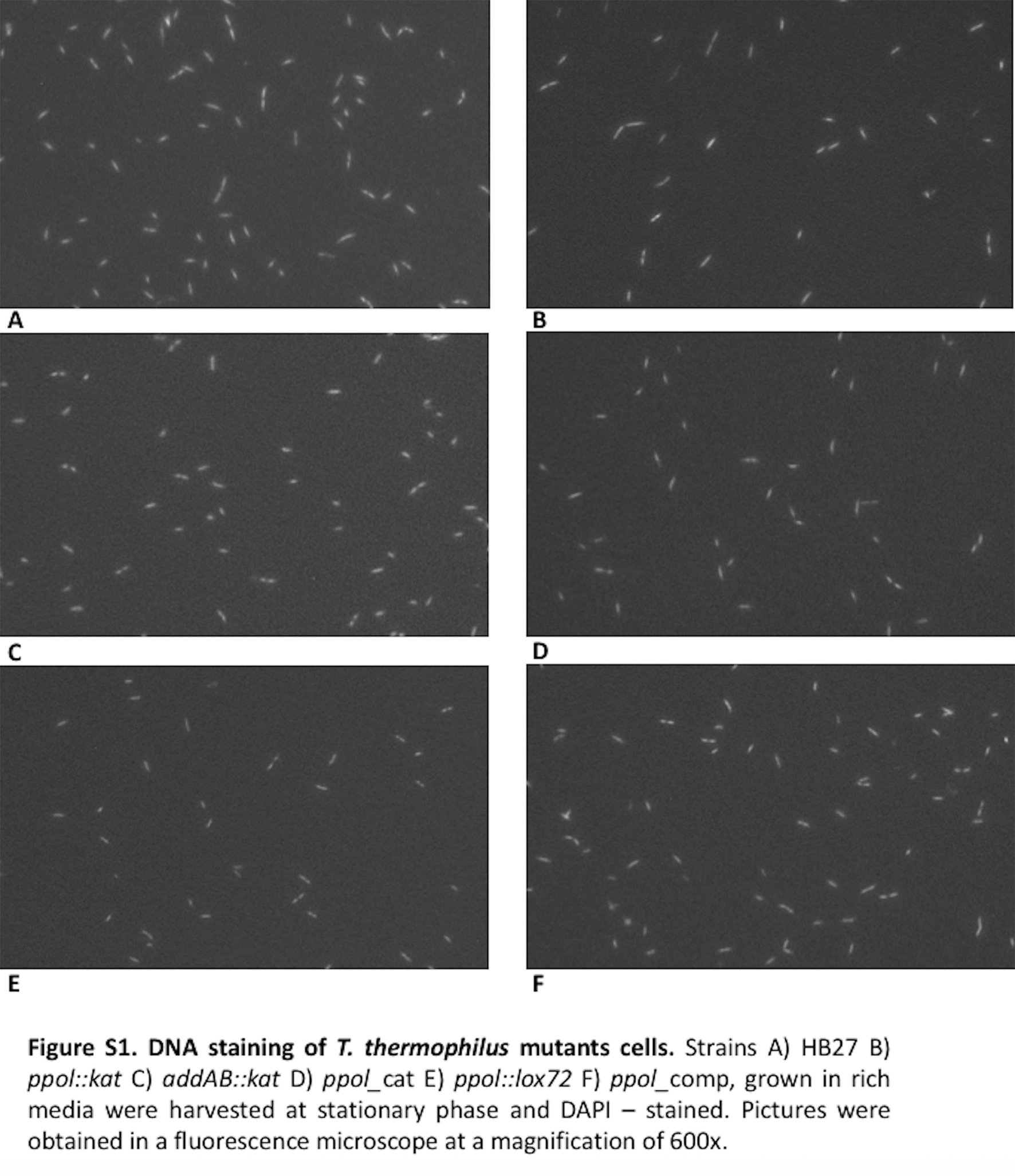

Supplement: Supplementary file 1 [file Image_1.PNG]
