# Supplementary material for: Deletion of the primase-polymerases encoding gene, located in a mobile element in Thermus thermophilus HB27, leads to loss of function mutation of addAB genes
Source: Front Microbiol. 2022 Dec 1;13:1005862. doi: 10.3389/fmicb.2022.1005862 (PMC9751324; doi:10.3389/fmicb.2022.1005862)
Supplement: Supplementary file 6 [file Table_5.DOCX]

Table S1. List of genes affected by the 13 473 bp chromosomic deletion in *ppol::lox72* and *ppol_*comp.

Table S2. List of variants of the three sequenced ppol::Kn strains respect to the HB27 lab stock. 0 denotes no variation, 1, as indicated. For the intergenic variants, the number of bp from the mutation to the nearest downstream starting codon are indicated.In the upper part are the chromososmal variants and in the lower part the pTT27 megaplasmid variants. Shadowed in orange are variants detected in the same gene for the three strains. Shadowed in red the common addA mutation.

Table S3. List of variants of the seven sequenced mutant strains respect to the HB27 lab stock. Indications as for Table S2.

Table S4. Thermus species with predicted proteins that show significant scores in BLASTp alignment (https://blast.ncbi.nlm.nih.gov/) to the indicated Tth HB27 proteins, against protein sequences from Thermus genus (taxid:270). AddA, AddB and Ppol protein sequences were used in searches, as well as RpoB (beta subunit of the RNA polymerase) as a control of an essential conserved protein. The one strain retrieved when searching with AddA but not with AddB is indicated, as well as, reciprocally, two retrieved with AddB and not with AddA, and the list of strains not retrieved with Ppol but appearing in Add or B sets is shown. RpoB retrieved all the strains obtained with AddA or B, plus, additionally, one that had neither (*T. islandicus*).
