# Supplementary material for: Deletion of the primase-polymerases encoding gene, located in a mobile element in Thermus thermophilus HB27, leads to loss of function mutation of addAB genes
Source: Front Microbiol. 2022 Dec 1;13:1005862. doi: 10.3389/fmicb.2022.1005862 (PMC9751324; doi:10.3389/fmicb.2022.1005862)
Supplement: Supplementary file 7 [file Table_6.DOCX]

**Supplementary discussion**

The genes coding for polyphosphate kinase (*ppk*) and phosphatase (*ppx*) are among the genes that form part of the 13 Kb deletion ocurring in *ppol*::lox72 and *ppol*_comp. It has been shown that polyphosphate production by Ppk regulates many types of stress response, and Ppk activity is counteracted by Ppx by degradation of polyphosphate (Brown and Kornberg, 2008). Interestingly, *ppol*::lox72 and *ppol*_comp do not appear to be very sensitive to different DNA damaging agents and show normal viability. We do not know if some of the additional mutations in these strains could compensate for the *ppK/ppX* loss.

Other mutations found in the sequenced genomes:

- Predicted change Leu442-Pro in *recG*. Present in *ppol*::lox72 and its derivative *ppol*_comp. RecG is a DNA helicase with proposed role in rescue of stalled replication forks by regression leading to a Holiday junction structure (Bianco and Lyubchenko, 2017). The equivalent of HB27 Leu442 in *E. coli* RecG (Ile367) is located at the end of an α-helix in domain 2 of the protein (Singleton et al., 2001). There is no information of the effect of mutants in that position.

- C to T change 39 bp upstream of *dnaB*. DnaB is the replicative DNA helicase, an essential gene. The change C to T bp at 39 from the initiation codon could affect the TATA box of the promoter increasing its strenght.

- G to A change 86 bp upstream of *pilA4* gene. Present in *addAB*::Kn and offspring. PilA is a major component of the pilus and it is essential for natural competence of the strain (Schwarzenlander and Averhoff, 2006). The mutation is not likely to be near the -35 box of the promoter, but still could affect some regulatory element.

- All the strains have point missense mutations in *TT_C1342*. This gene codes for a putative divalent heavy-metal cations transporter. The mutations are different for different families of mutants, the *addAB* derivative plus *ppol*_cat has the change Ile138Thr, the *ppol*::lox72 and derivatives, Thr114Ala, and *ppol*::Kn, Arg160Gln.

- Mutation leading to a frameshift in Arg165 of the heat-inducible transcription repressor HrcA. Occurring in *ppol*::lox72 and its derivative. HrcA prevents heat-shock induction of class I heat shock genes (Inoue et al., 2012). The loss of function of this protein would lead to increased expression of heat shock genes.

- The rest of the mutations cannot be easily connected with the phenotypes and routes we are studying.

- The same mutation producing the change Ala311-frameshift in the *addA* gene has ocurred in four strains independently *ppol*::Kn, *ppol*::KnR1, *ppol*::KnR4 and *ppol*_cat, since they are not derived from one another and the parental HB27 does not have the mutation. A possible explanation for this is that this particular position in the genome is prone to mutate, and, in the absence of Ppol, the mutation would not be corrected and it would give raise to loss of function of AddAB that will be selected as it compensates the loss of *ppol*.

**References**

Bianco, P. R., and Lyubchenko, Y. L. (2017). SSB and the RecG DNA helicase: an intimate association to rescue a stalled replication fork: SB and the RecG DNA Helicase. *Protein Science* 26, 638–649. doi: 10.1002/pro.3114.

Brown, M. R. W., and Kornberg, A. (2008). The long and short of it – polyphosphate, PPK and bacterial survival. *Trends in Biochemical Sciences* 33, 284–290. doi: 10.1016/j.tibs.2008.04.005.

Inoue, M., Mitarai, N., and Trusina, A. (2012). Circuit architecture explains functional similarity of bacterial heat shock responses. *Phys. Biol.* 9, 066003. doi: 10.1088/1478-3975/9/6/066003.

Schwarzenlander, C., and Averhoff, B. (2006). Characterization of DNA transport in the thermophilic bacterium Thermus thermophilus HB27. *FEBS Journal* 273, 4210–4218. doi: 10.1111/j.1742-4658.2006.05416.x.

Singleton, M. R., Scaife, S., and Wigley, D. B. (2001). Structural Analysis of DNA Replication Fork Reversal by RecG. *Cell* 107, 79–89. doi: 10.1016/S0092-8674(01)00501-3.
